# Performance of Double Reading Mammography in an Iranian Population and Its Effect on Patient Outcome

**DOI:** 10.5812/iranjradiol.11729

**Published:** 2013-05-20

**Authors:** Maryam Moradi, Kobra Ganji, Niloufar Teyfouri, Farzaneh Kolahdoozan

**Affiliations:** 1 Department of Radiology, Isfahan University of Medical Sciences, Isfahan, Iran; 2 Atieh Imaging Center, Isfahan, Iran; 3 Medical Image and Signal Processing Research Center, Isfahan University of Medical Sciences, Isfahan, Iran

**Keywords:** Double Reading, Mammography, Recall Rate

## Abstract

**Background:**

Considering the importance and responsibility of reporting mammography and the necessity to notice details with a high degree of precision, double reading mammography has been introduced and recommended.

**Objectives:**

This study aimed to assess the performance of double reading of mammograms and its effect on patient outcomes.

**Patients and Methods:**

Throughout this cross sectional study, 1284 digitized mammographic views of 642 breasts which belonged to 339 women (of which 303 were bilateral and 36 were unilateral mammographies) were enrolled. Two independent radiologists interpreted these mammograms and BI-RADS categories of both reports were compared. Discordant results were determined and assumed significant if they were in the positive (BI-RADS 0, 4, 5) versus negative (BI-RADS 1, 2, 3) groups and then significant discordant cases were followed up to determine benign versus malignant final diagnosis. The recall rate was calculated for each reader. Inter-observer agreement in breast density was determined by Kappa test.

**Results:**

Readers had consensus on BI-RADS categories in 459 breasts (71%), but diverse categories were used for 183 breasts (29%), including 132 significant and 51 non-significant discrepancies. According to weighted Kappa test, agreement between two readers in positive or negative reports was 0.78 (95% CI=0.73-0.83) and in parenchymal density, it was 0.73 (95% CI=0.7-0.77). Most of the discrepancies were between category zero versus categories 1 and 2 (63.4%). The recall rate was 36% for the first and 44% for the second reader. Among 132 significant discordant results, one case had the final diagnosis of malignancy and the others had benign or negative diagnosis. There was 0.2% increase in cancer detection rate by double reading.

**Conclusion:**

This study shows no significant improvement in the cancer detection rate by double reading; however, a lower recall rate could be a more helpful consequence.

## 1. Background

Breast cancer is a common malignancy worldwide and the most common cancer of women in Iran (1, 2). Mammography aims to detect cancer in asymptomatic women when it is easier to cure and remains the cornerstone of population-based breast cancer screening. Considering the importance of the report in mammography and the necessity to notice details with a high degree of precision, double reading or more than one reader for mammography was proposed in 1991 (3). Double reading has been recommended as a routine or standard protocol by some guidelines and studies (3-5); however, subsequent improvement in cancer detection rate varies greatly between different studies (6). Although the efficacy of second reading has been confirmed by some studies (7, 8), others focused on the limited number of additional cancer detection (9), decrease in the positive predictive value and increase in the recall rate and anxiety (10-12). Apart from this, the cost effectiveness of this method is under doubt (11, 13, 14) and finally further research was recommended to assess relative benefits from double reading and to estimate the impact on patient outcomes (15). Regarding the different prevalence and incidence of breast cancer in each population and diverse mammographic interpretation approach due to different training and forensic aspects, the effectiveness of double reading could be different for each country. To our knowledge, until now, double reading has not been studied among the Iranian population.

## 2. Objectives

This study aimed to describe the results of double reading of mammograms and to assess its effect on changing the final report (between negative and positive), to focus on the effect of discrepancy on the final patient outcome (benign to malignant and vice versa) and improvement in the cancer detection rate.

## 3. Patients and Methods

This cross sectional study was approved by the ethics committee of Isfahan University of Medical Sciences (project number 289230). Digitized mammograms of women who attended for both diagnostic and screening purposes in 2008 and 2009, were collected from the database of Medical Image and Signal Processing Research Center (MISP) of Isfahan University of Medical Sciences. Two independent radiologists with 8 and 10 years experience in breast imaging, interpreted these mammograms. Each breast of a woman has its own characteristics, so readers assessed them separately (16). Findings of each breast were described by two readers who were blind to each other’s reports and finally reported according to the Breast Imaging Reporting and Data System (BI-RADS) as categories 0 to 5 (17). Breast density was described according to the BI-RADS lexicon and classified to almost entirely fat (F), scattered fibroglandular densities (SFGD), heterogeneously dense (HD) and extremely dense (ED) (17). The frequency of positive results (BI-RADS category 0, 4, 5) and negative results (BI- RADS category 1, 2, 3) were determined for each reader (18). The recall rate was defined as the proportion of individuals recalled for additional work-up and calculated as the ratio of positive (BI-RADS 0,4,5) to all (BIRADS 0-5) reports. The BI-RADS categories of the two reports were compared for each breast and discordant results were determined. Discrepancy was assumed as "significant" if reports were positive (categories 0, 4, 5) against negative (category 1-3) and "non significant" if reports were in same (positive or negative) groups, according to the reader’s opinion (19). Agreement of the two observers for the type of breast density and also for positive against negative reports were evaluated using SPSS software version 20, and presented as Kappa values. Perfect agreement is indicated as a Kappa value of 1.0, and a Kappa value of 0 means no agreement. Kappa values less than 0.20 mean slight agreement; 0.21-0.40, fair agreement; 0.41-0.60, moderate agreement; 0.61-0.80, substantial agreement; and 0.81-0.99, almost perfect agreement between observers. To evaluate the effect of double reading on the patient outcome, women with significant discordant reports were followed up to identify benign against malignant results. Definite diagnosis was made either on pathologic results of breast tissue sampling or upon the two-year later imaging results (20).

## 4. Results

1284 mammographic views of 642 breasts were enrolled that belonged to 339 women (303 with bilateral and 36 with unilateral mammography). The mean age of participants was 47.7 (range: 30 to 76) years. Distribution of breast parenchymal density based on the radiologists’ opinions is shown in (Table 1).

**Table 1.** Frequency of Different Types of Parenchymal Density in the Evaluated Women

	F ^a^	SFGD	HD	ED	Total
First Reader, No. (%)	105 (31)	128 (38)	99 (29)	7 (2)	339 (100)
Second Reader, No. (%)	91 (27)	124 (36)	115 (34)	9 (3)	339 (100)

^a^Almost entirely fatty

Abbreviations: SFGD, Scattered fibroglandular density; HD, Heterogenously dense; ED, Extremely dense

According to weighted Kappa test, the agreement between the two readers was 0.59 (95% CI=0.57-0.61) for fatty type breast parenchyma (F), 0.88 (95% CI=0.86-0.90) for SFGD, 0.53 (95% CI=0.51-0.55) for HD and 0.94 (95% CI=0.86-1) for ED tissue type. The overall agreement in breast density type was 0.74 (95% CI=0.70-0.77).The frequency of different BI-RADS categories for both readers are shown in (Table 2).

**Table 2.** Frequency of Different BI-RADS Categories in the Evaluated Breasts

BI-RADS Categories	0	1	2	3	4	5	Total
First Reader, No. (%)	200 (31.2)	278 (43.3)	115 (17.9)	17 (2.6)	22 (3.4)	10 (1.6)	642 (100)
Second Reader, No. (%)	255 (39.7)	275 (42.8)	67 (10.5)	18 (2.8)	16 (2.5)	11 (1.7)	642 (100)

Reports of first readers were positive for 232 (36%) and negative for 410 (64%) breasts; however, these result for the second readers were 282 (44%) and 360 (56%), respectively. According to weighted Kappa test, the agreement between the two readers on positivism or negativism of the reports was 0.78 (95% CI=0.73-0.83). Readers had consensus on the BI-RADS categorization in 459 breasts (71%), but diverse categories were used for 183 breasts (29%) including 132 (21%) "significant" and 51 (9%)"non-significant" difference. Discordant BI-RADS categories were categorized in ten groups, their frequency with detailed related findings are shown in (Table 3).

**Table 3.** BI-RADS Discrepancies, Distribution and Related Causes

Type of Discrepancy	No. (%)	Related Causes and Findings
Category 0 vs. 1 ^a^	88 (48.1)	Focal density detected only by one reader=78 cases
		Mass detected only by one reader=8 cases
		Zero category is used only because extremely dense breast=2 cases
Category 0 vs. 2 ^a^	28 (15.3)	Intra mammary LN by one reader is considered as focal asymmetric density by another=4 cases
		In addition to benign findings, focal density is noted by one reader=24 cases
Category 0 vs. 3 ^a^	13 (7.1)	Same findings are considered by both readers, but different categories are used
Category 0 vs. 4	7 (3.8)	Same findings are considered by both readers, but different categories are used
Category 0 vs. 5	1 (0.6)	Same finding are considered by both readers but different categories are used
Category 1 vs. 2	30 (16.4)	Intra mammary LN is noted only by one reader =7 cases
		Benign calcifications or benign microcalcifications are considered only by one of the readers=23 cases
Category 1 vs. 3	3 (1.6)	Probably benign mass detected by one reader =2 cases
		Probably benign microcalcification detected by one reader=1 case
Category 1 vs. 4^a^	3 (1.6)	Suspicious mass detected by one reader=2cases
		Suspicious microcalcification detected by one reader=1 case
Category 2 vs. 3	6 (3.3)	Intramammary LN by one reader is considered as probably benign mass by another reader=5 cases
		In addition to benign finding, a probably benign density is detected by another reader =1 case
Category 4 vs. 5	4 (2.2)	Same findings are considered by both readers, but different categories are used

^a^Significant discrepancy

Abbreviation: LN, Lymph node

We had no discrepancy between category 5 versus categories 0-3 and also category 4 versus categories 1-3. The most common type of significant discordant results was category zero against 1 and zero against two, which were mostly related to focal asymmetric density and was noticed only by one reader. Women who had significant discordant reports (132 cases) were followed according to pathologic results (in 28 women) and two-year imaging results (in 104 women). The final diagnosis of the followed women was benign in 131 (99%) and malignant in one (1%) of the cases; therefore, improvement in the cancer detection rate by double reading was 0.2%. The recall rate was 36% for the first reader and 44% for the second reader.

## 5. Discussion

According to this study, the inter-observer agreement in breast density type is good (substantial agreement) though not perfect. Previous studies that assessed inter-observer variability showed moderate agreement such as the study carried out by Berg and co-authors (Kappa=0.43) (21), and another study performed by Ciatto et al. (Kappa=0.54) (22), or higher agreement, as a study conducted by Ooms et al. (Kappa=0.77) (23). Our study is comparable with the last mentioned study (Kappa=0.74). This improvement could be due to more education, as was also mentioned by Ooms (23). Furthermore, D’Orsi et al. recommended some modification in the defined percentages of some density types, for example almost entire fat would be up to 10% density instead of 0-25% and scattered fibroglandular densities might then range from 11-50%, instead of 25-50% (18), so tissue type discrepancy in this study could be justifiable. Double reading of 642 breast mammograms in this study resulted in only one more detected malignancy or 0.2% increase in the cancer detection rate (CDR) that is significantly lower than improvement in the cancer detection rate of previous studies (7, 12, 24-27). In addition, the readers’ agreement in this study on the final report of positive or negative is lower than the comparative study by Duijm (20). This can be due to limitation in the number of cases and readers. More numerous cases and more readers may cause different results. On the other hand, Beam et al. believed that expected gain in true-positive results (TPR) in double reading studies depends on the experience of the radiologists (28). More improvement in TPR may be achieved by repeating the reading by more experienced radiologists, so another cause of no significant improvement in CDR in this study may be due to the similarity of the reader’s experience. The most common type of significant discrepancy in BI-RADS categories was category 0 versus 1 (64%) and 0 versus 2 (23%), which were mostly related to focal asymmetric densities and were mentioned only by one of the readers. It is important to note that all these focal asymmetries that were mentioned by only one reader were related to nonspecific or benign findings in the follow-up and none of them were related to significant or malignant pathologies. The recall rate of both readers in this study was significantly higher than the suitable or target recall rate (19), and this could be another cause of less improvement in CDR by double reading in this study. As we know, a higher recall rate is related with more false positive results, more anxiety and cost. Although a higher recall rate for this study could be partly due to mixed diagnostic and screening purposes instead of pure screening purpose, this is still higher than the optimal recall rate and one of the most important aspects of this study could be the idea of lowering the recall rate by double reading in our practice. Based on this idea, we can recall a patient when both readers’ agreement is achieved and therefore we may expect a less recall rate and related anxiety and cost for patients. Another detected cancer by double reading in this study was related to architectural distortion which was detected only by one of the radiologists and this is similar to a previous study conducted by Cornford et al. (25). Finally, further studies with more readers and more cases with pure screening mammograms are recommended. In addition, further studies are necessary on the evaluation of recall rate in Iran and if the recall rate is higher than optimum (as expected), lowering the recall rate might be a more important consequence of double reading in our practice. This study shows no significant improvement in the cancer detection rate by double reading; however a lower recall rate could be a more helpful consequence.
